# 13c-(2-Chloro­ethoxy)-1,13c-dihydro-2,3-epoxy­dibenzo[*a*,*kl*]xanthan-1-one

**DOI:** 10.1107/S1600536808030973

**Published:** 2008-10-04

**Authors:** Jin-Xiang Chen, Yu-Qin Wang, Shu-Guang Wu, Zhi-Hong Jiang, Zhi-Peng Chen

**Affiliations:** a School of Pharmaceutical Science, Southern Medical University, Guangzhou 510515, Guangdong, People’s Republic of China

## Abstract

The title compound, C_22_H_15_ClO_4_, containing three chiral C atoms, is an inter­mediate in the design of chiral alcohols. In the crystal structure, a chain structure is generated through C—H⋯O contacts and an intramolecular C—H⋯O interaction also occurs. The dihedral angle between the benzene ring and the naphthalene system is 16.5°.

## Related literature

For related literature, see: Aronne *et al.* (2008 ▶); Sasidharan *et al.* (2002 ▶); Tan *et al.* (2001 ▶); Wang *et al.* (2003 ▶); Yamazaki (2008 ▶).
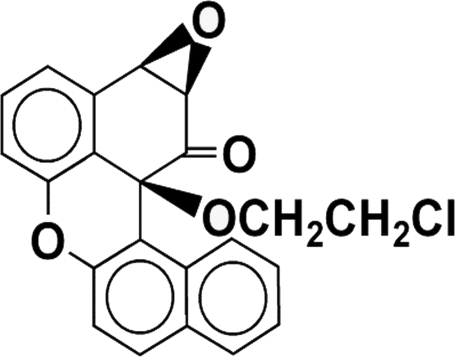


## Experimental

### 

#### Crystal data

C_22_H_15_ClO_4_
*M* *_r_* = 378.79
Orthorhombic, 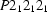

*a* = 7.7966 (13) Å
*b* = 10.4468 (18) Å
*c* = 21.349 (4) Å
*V* = 1738.9 (5) Å^3^
*Z* = 4
Mo *K*α radiation
μ = 0.25 mm^−1^
*T* = 193 (2) K
0.30 × 0.20 × 0.10 mm

#### Data collection

Rigaku Mercury diffractometer
Absorption correction: multi-scan (Jacobson, 1998 ▶) *T*_min_ = 0.930, *T*_max_ = 0.966
10185 measured reflections
3416 independent reflections
2809 reflections with *I* > 2σ(*I*)
*R*_int_ = 0.029

#### Refinement

*R*[*F* ^2^ > 2σ(*F* ^2^)] = 0.047
*wR*(*F* ^2^) = 0.136
*S* = 1.06
3416 reflections
244 parameters
19 restraints
H-atom parameters constrained
Δρ_max_ = 0.59 e Å^−3^
Δρ_min_ = −0.35 e Å^−3^
Absolute structure: Flack (1983 ▶) , 1440 Friedel pairs
Flack parameter: 0.00 (11)

### 

Data collection: *CrystalClear* (Rigaku/MSC, 2001 ▶); cell refinement: *CrystalClear*; data reduction: *CrystalStructure* (Rigaku/MSC, 2004 ▶); program(s) used to solve structure: *SHELXS97* (Sheldrick, 2008 ▶); program(s) used to refine structure: *SHELXL97* (Sheldrick, 2008 ▶); molecular graphics: *ORTEPII* (Johnson, 1976 ▶); software used to prepare material for publication: *SHELXL97*.

## Supplementary Material

Crystal structure: contains datablocks global, I. DOI: 10.1107/S1600536808030973/tk2302sup1.cif

Structure factors: contains datablocks I. DOI: 10.1107/S1600536808030973/tk2302Isup2.hkl

Additional supplementary materials:  crystallographic information; 3D view; checkCIF report

## Figures and Tables

**Table 1:** Hydrogen-bond geometry (Å, °)

*D*—H⋯*A*	*D*—H	H⋯*A*	*D*⋯*A*	*D*—H⋯*A*
C14—H14*A*⋯O2^i^	0.95	2.55	3.392 (4)	147
C19—H19*A*⋯O4	0.95	2.54	3.084 (3)	117

Symmetry code: (i) 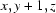.

**Fig. 1.**
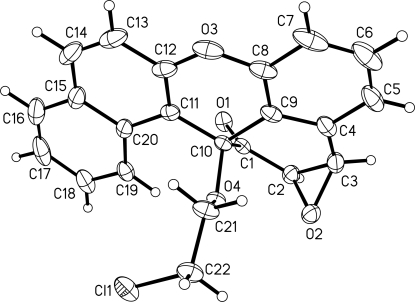
Molecular structure of (I) showing atom labelling and 30% probability ellipsoids. Hydrogen atoms are drawn as spheres of arbitrary radii.

**Fig. 2.**
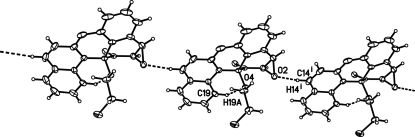
One-dimensional chain aligned along the b axis in (I) consolidated by C-H···O contacts, shown as dashed lines.

## supplementary crystallographic information

### Comment

Epoxides are well known as one of the most valuable building blocks used as intermediates and precursors for pharmaceuticals (Yamazaki, 2008; Aronne, *et al.*, 2008). The title compound, (I), is a key intermediate in the preparation of chiral alcohols, which we are designing for potential use as antiviral agents. The structure of (I), Fig. 1, provides information on the potential stereoselectivity of its ring-opening reactions (Sasidharan *et al.*, 2002; Wang *et al.*, 2003). The molecule of (I) contains six fused rings with the three aromatic rings almost coplanar. The six-membered carbocyclic ring adopts a slightly twisted boat conformation and the pyran ring is nearly planar. The epoxy group points in the same direction as the OCH_2_CH_2_Cl group, having a *syn* relationship. In the crystal structure, molecules of (I) associate in a head-to-tail manner, parallel to the *b* axis, *via* O-H···O hydrogen bonds to form a 1D structure, Fig. 2 and Table 1.

### Experimental

Compound (I) was obtained by epoxidation of 13*c*-(2-chloroethyloxy)-1-oxo-1,13*c*-dihydrodibenzo[a,kl]xanthene in methanol with aqueous hydrogen peroxide (30%) under mild reaction conditions (Tan *et al.*, 2001). Compound (I) was the main product, isolated in a yield of 92%. Crystals suitable for X-ray analysis were obtained from the slow evaporation of an acetone solution (m.p. 527–529 K). IR (KBr disk): 3409, 2905, 2359, 1720 (s, C=O), 1454, 1267, 1097, 776, 755, 508 cm^-1^. ^1^H NMR (300 MHz in CDCl_3_/TMS): 3.15–3.25 (m, 2H), 3.27–3.38 (m, 2H), 4.04 (d, J = 3.9 Hz, 1H), 4.34 (d, J = 3.9 Hz, 1H), 7.24–7.26 (m, 1H), 7.27 (d, J = 9.0 Hz, 1H), 7.34–7.49 (m, 4H), 7.78–7.79 (m, 1H), 7.86 (d, J = 9.0 Hz, 1H), 7.96–8.02 (m, 1H) p.p.m. FAB-MS (m/*z*): 299(*M*^+^ –OCH_2_CH_2_Cl).

### Refinement

The hydrogen atoms were placed in geometrically idealized positions with C—H = 0.95 - 1.00 Å and constrained to ride on their parent atoms with *U*_iso_(H) = 1.2*U*_eq_(C).

### Crystal data

**Table:**

C_22_H_15_ClO_4_	*F*(000) = 784
*M**_r_* = 378.79	*D*_x_ = 1.447 Mg m^−^^3^
Orthorhombic, *P*2_1_2_1_2_1_	Mo *K*α radiation, λ = 0.71073 Å
Hall symbol: P 2ac 2ab	Cell parameters from 3462 reflections
*a* = 7.7966 (13) Å	θ = 3.0–26.0°
*b* = 10.4468 (18) Å	µ = 0.25 mm^−^^1^
*c* = 21.349 (4) Å	*T* = 193 K
*V* = 1738.9 (5) Å^3^	Block, white
*Z* = 4	0.30 × 0.20 × 0.10 mm

### Data collection

**Table:**

Rigaku Mercury diffractometer	3416 independent reflections
Radiation source: fine-focus sealed tube	2809 reflections with *I* > 2σ(*I*)
graphite	*R*_int_ = 0.029
ω scans	θ_max_ = 26.0°, θ_min_ = 1.9°
Absorption correction: multi-scan Jacobson (1998)	*h* = −9→9
*T*_min_ = 0.930, *T*_max_ = 0.966	*k* = −12→12
10185 measured reflections	*l* = −19→26

### Refinement

**Table:**

Refinement on *F*^2^	Secondary atom site location: difference Fourier map
Least-squares matrix: full	Hydrogen site location: inferred from neighbouring sites
*R*[*F*^2^ > 2σ(*F*^2^)] = 0.047	H-atom parameters constrained
*wR*(*F*^2^) = 0.136	*w* = 1/[σ^2^(*F*_o_^2^) + (0.074*P*)^2^ + 0.4256*P*] where *P* = (*F*_o_^2^ + 2*F*_c_^2^)/3
*S* = 1.06	(Δ/σ)_max_ < 0.001
3416 reflections	Δρ_max_ = 0.59 e Å^−^^3^
244 parameters	Δρ_min_ = −0.35 e Å^−^^3^
19 restraints	Absolute structure: (Flack, 1983)
Primary atom site location: structure-invariant direct methods	Flack parameter: 0.00 (11)

### Special details

**Table:**

Geometry. All e.s.d.'s (except the e.s.d. in the dihedral angle between two l.s. planes) are estimated using the full covariance matrix. The cell e.s.d.'s are taken into account individually in the estimation of e.s.d.'s in distances, angles and torsion angles; correlations between e.s.d.'s in cell parameters are only used when they are defined by crystal symmetry. An approximate (isotropic) treatment of cell e.s.d.'s is used for estimating e.s.d.'s involving l.s. planes.
Refinement. Refinement of *F*^2^ against ALL reflections. The weighted *R*-factor *wR* and goodness of fit *S* are based on *F*^2^, conventional *R*-factors *R* are based on *F*, with *F* set to zero for negative *F*^2^. The threshold expression of *F*^2^ > σ(*F*^2^) is used only for calculating *R*-factors(gt) *etc*. and is not relevant to the choice of reflections for refinement. *R*-factors based on *F*^2^ are statistically about twice as large as those based on *F*, and *R*- factors based on ALL data will be even larger.

### Fractional atomic coordinates and isotropic or equivalent isotropic displacement parameters (Å^2^)

**Table:**

	*x*	*y*	*z*	*U*_iso_*/*U*_eq_	
Cl1	0.46182 (15)	0.38912 (11)	0.01032 (4)	0.0875 (3)	
O1	−0.1176 (2)	0.3230 (2)	−0.16123 (9)	0.0532 (5)	
O2	0.2117 (3)	0.0809 (2)	−0.15674 (11)	0.0644 (6)	
O3	0.3149 (3)	0.5535 (3)	−0.24976 (11)	0.0751 (7)	
O4	0.2976 (2)	0.31516 (16)	−0.11496 (8)	0.0407 (4)	
C1	0.0243 (3)	0.2781 (2)	−0.16079 (11)	0.0405 (5)	
C2	0.0484 (4)	0.1375 (3)	−0.16874 (14)	0.0539 (7)	
H2A	−0.0548	0.0821	−0.1626	0.065*	
C3	0.1656 (4)	0.1034 (3)	−0.22079 (15)	0.0639 (9)	
H3A	0.1337	0.0264	−0.2460	0.077*	
C4	0.2524 (4)	0.2067 (4)	−0.25451 (14)	0.0615 (8)	
C5	0.3171 (5)	0.1828 (5)	−0.31363 (17)	0.0876 (14)	
H5A	0.3128	0.0983	−0.3300	0.105*	
C6	0.3871 (5)	0.2784 (7)	−0.34885 (19)	0.1044 (19)	
H6A	0.4346	0.2595	−0.3888	0.125*	
C7	0.3890 (5)	0.4024 (6)	−0.32672 (18)	0.0925 (15)	
H7A	0.4359	0.4696	−0.3513	0.111*	
C8	0.3208 (4)	0.4275 (4)	−0.26739 (15)	0.0637 (8)	
C9	0.2571 (3)	0.3325 (3)	−0.22965 (13)	0.0503 (7)	
C10	0.1881 (3)	0.3613 (2)	−0.16413 (11)	0.0398 (5)	
C11	0.1543 (3)	0.5025 (3)	−0.15546 (13)	0.0467 (6)	
C12	0.2211 (4)	0.5882 (3)	−0.19788 (16)	0.0621 (9)	
C13	0.1977 (6)	0.7205 (4)	−0.1912 (2)	0.0874 (14)	
H13A	0.2446	0.7775	−0.2213	0.105*	
C14	0.1084 (6)	0.7670 (4)	−0.1419 (3)	0.0954 (17)	
H14A	0.0913	0.8567	−0.1382	0.114*	
C15	0.0399 (5)	0.6845 (3)	−0.09575 (19)	0.0747 (11)	
C16	−0.0529 (6)	0.7314 (5)	−0.0437 (3)	0.0975 (16)	
H16A	−0.0749	0.8206	−0.0407	0.117*	
C17	−0.1110 (5)	0.6531 (6)	0.0017 (3)	0.1038 (18)	
H17A	−0.1753	0.6875	0.0355	0.125*	
C18	−0.0773 (4)	0.5204 (4)	−0.00048 (18)	0.0763 (11)	
H18A	−0.1155	0.4656	0.0322	0.092*	
C19	0.0119 (4)	0.4717 (3)	−0.05086 (14)	0.0554 (7)	
H19A	0.0361	0.3827	−0.0523	0.067*	
C20	0.0683 (4)	0.5505 (3)	−0.10016 (15)	0.0512 (7)	
C21	0.4673 (3)	0.3628 (3)	−0.11653 (14)	0.0581 (8)	
H21A	0.4656	0.4573	−0.1196	0.070*	
H21B	0.5282	0.3285	−0.1536	0.070*	
C22	0.5569 (4)	0.3234 (4)	−0.05865 (15)	0.0674 (9)	
H22A	0.6781	0.3511	−0.0610	0.081*	
H22B	0.5555	0.2288	−0.0557	0.081*	

### Atomic displacement parameters (Å^2^)

**Table:**

	*U*^11^	*U*^22^	*U*^33^	*U*^12^	*U*^13^	*U*^23^
Cl1	0.0914 (7)	0.1130 (8)	0.0581 (5)	−0.0041 (6)	−0.0129 (5)	−0.0133 (5)
O1	0.0330 (9)	0.0667 (12)	0.0600 (12)	−0.0019 (9)	−0.0022 (8)	−0.0140 (10)
O2	0.0653 (14)	0.0560 (11)	0.0719 (14)	0.0091 (10)	−0.0164 (11)	−0.0079 (10)
O3	0.0592 (12)	0.1059 (17)	0.0602 (13)	−0.0229 (13)	−0.0064 (11)	0.0374 (12)
O4	0.0337 (8)	0.0517 (9)	0.0366 (9)	−0.0029 (7)	−0.0054 (7)	0.0054 (8)
C1	0.0370 (13)	0.0516 (13)	0.0329 (12)	−0.0024 (11)	−0.0016 (10)	−0.0019 (10)
C2	0.0519 (15)	0.0492 (15)	0.0606 (17)	−0.0034 (13)	−0.0072 (14)	−0.0061 (13)
C3	0.0591 (19)	0.0673 (19)	0.065 (2)	0.0146 (16)	−0.0136 (15)	−0.0207 (17)
C4	0.0418 (14)	0.098 (2)	0.0451 (15)	0.0212 (16)	−0.0067 (12)	−0.0141 (16)
C5	0.057 (2)	0.154 (4)	0.052 (2)	0.038 (2)	−0.0018 (17)	−0.028 (2)
C6	0.056 (2)	0.207 (6)	0.050 (2)	0.024 (3)	0.0092 (17)	−0.007 (3)
C7	0.0469 (19)	0.177 (5)	0.053 (2)	−0.004 (2)	0.0046 (15)	0.037 (3)
C8	0.0410 (14)	0.101 (2)	0.0493 (15)	−0.0078 (16)	−0.0034 (13)	0.0233 (16)
C9	0.0310 (12)	0.0801 (19)	0.0399 (13)	0.0032 (13)	−0.0049 (10)	0.0050 (14)
C10	0.0349 (12)	0.0495 (13)	0.0349 (12)	−0.0024 (10)	−0.0053 (10)	0.0018 (10)
C11	0.0391 (13)	0.0501 (14)	0.0509 (15)	−0.0063 (11)	−0.0139 (11)	0.0050 (12)
C12	0.0526 (18)	0.0618 (18)	0.072 (2)	−0.0124 (15)	−0.0249 (16)	0.0204 (16)
C13	0.088 (3)	0.057 (2)	0.117 (3)	−0.023 (2)	−0.052 (3)	0.032 (2)
C14	0.099 (3)	0.0414 (17)	0.146 (4)	−0.004 (2)	−0.069 (3)	0.004 (2)
C15	0.067 (2)	0.0560 (18)	0.101 (3)	0.0121 (17)	−0.045 (2)	−0.0239 (19)
C16	0.082 (3)	0.080 (3)	0.131 (4)	0.031 (2)	−0.044 (3)	−0.053 (3)
C17	0.063 (2)	0.142 (4)	0.107 (4)	0.032 (3)	−0.021 (2)	−0.079 (3)
C18	0.0557 (18)	0.109 (3)	0.064 (2)	0.0057 (19)	−0.0051 (15)	−0.034 (2)
C19	0.0439 (14)	0.0697 (18)	0.0527 (17)	−0.0014 (13)	−0.0030 (12)	−0.0148 (14)
C20	0.0399 (14)	0.0498 (14)	0.0638 (18)	0.0016 (12)	−0.0183 (13)	−0.0121 (13)
C21	0.0337 (13)	0.092 (2)	0.0481 (15)	−0.0082 (14)	−0.0058 (12)	0.0153 (15)
C22	0.0472 (16)	0.094 (2)	0.0613 (19)	0.0008 (17)	−0.0121 (15)	0.0086 (17)

### Geometric parameters (Å, °)

**Table:**

Cl1—C22	1.786 (4)	C10—C11	1.509 (4)
O1—C1	1.201 (3)	C11—C12	1.376 (4)
O2—C2	1.427 (4)	C11—C20	1.448 (4)
O2—C3	1.433 (4)	C12—C13	1.401 (5)
O3—C8	1.370 (5)	C13—C14	1.353 (7)
O3—C12	1.376 (4)	C13—H13A	0.9500
O4—C21	1.414 (3)	C14—C15	1.413 (6)
O4—C10	1.437 (3)	C14—H14A	0.9500
C1—C2	1.490 (4)	C15—C16	1.413 (7)
C1—C10	1.546 (3)	C15—C20	1.421 (4)
C2—C3	1.482 (4)	C16—C17	1.347 (7)
C2—H2A	1.0000	C16—H16A	0.9500
C3—C4	1.463 (5)	C17—C18	1.411 (7)
C3—H3A	1.0000	C17—H17A	0.9500
C4—C5	1.382 (5)	C18—C19	1.378 (5)
C4—C9	1.418 (5)	C18—H18A	0.9500
C5—C6	1.364 (7)	C19—C20	1.406 (5)
C5—H5A	0.9500	C19—H19A	0.9500
C6—C7	1.379 (8)	C21—C22	1.478 (4)
C6—H6A	0.9500	C21—H21A	0.9900
C7—C8	1.399 (5)	C21—H21B	0.9900
C7—H7A	0.9500	C22—H22A	0.9900
C8—C9	1.371 (4)	C22—H22B	0.9900
C9—C10	1.529 (4)		
			
C2—O2—C3	62.4 (2)	C12—C11—C20	119.1 (3)
C8—O3—C12	119.5 (2)	C12—C11—C10	119.3 (3)
C21—O4—C10	114.88 (19)	C20—C11—C10	121.3 (2)
O1—C1—C2	120.0 (3)	C11—C12—O3	124.0 (3)
O1—C1—C10	122.7 (2)	C11—C12—C13	121.7 (4)
C2—C1—C10	116.4 (2)	O3—C12—C13	114.3 (3)
O2—C2—C3	59.0 (2)	C14—C13—C12	120.1 (4)
O2—C2—C1	120.0 (2)	C14—C13—H13A	120.0
C3—C2—C1	113.6 (3)	C12—C13—H13A	120.0
O2—C2—H2A	117.0	C13—C14—C15	121.1 (3)
C3—C2—H2A	117.0	C13—C14—H14A	119.4
C1—C2—H2A	117.0	C15—C14—H14A	119.4
O2—C3—C4	118.3 (3)	C14—C15—C16	122.0 (4)
O2—C3—C2	58.6 (2)	C14—C15—C20	119.8 (4)
C4—C3—C2	118.5 (3)	C16—C15—C20	118.2 (4)
O2—C3—H3A	116.4	C17—C16—C15	121.8 (4)
C4—C3—H3A	116.4	C17—C16—H16A	119.1
C2—C3—H3A	116.4	C15—C16—H16A	119.1
C5—C4—C9	120.0 (4)	C16—C17—C18	120.7 (4)
C5—C4—C3	119.0 (4)	C16—C17—H17A	119.7
C9—C4—C3	120.7 (3)	C18—C17—H17A	119.7
C6—C5—C4	121.1 (5)	C19—C18—C17	118.8 (4)
C6—C5—H5A	119.4	C19—C18—H18A	120.6
C4—C5—H5A	119.4	C17—C18—H18A	120.6
C5—C6—C7	120.2 (4)	C18—C19—C20	121.8 (3)
C5—C6—H6A	119.9	C18—C19—H19A	119.1
C7—C6—H6A	119.9	C20—C19—H19A	119.1
C6—C7—C8	118.8 (4)	C19—C20—C15	118.6 (3)
C6—C7—H7A	120.6	C19—C20—C11	123.6 (2)
C8—C7—H7A	120.6	C15—C20—C11	117.9 (3)
O3—C8—C9	121.5 (3)	O4—C21—C22	108.9 (2)
O3—C8—C7	116.2 (4)	O4—C21—H21A	109.9
C9—C8—C7	122.3 (4)	C22—C21—H21A	109.9
C8—C9—C4	117.4 (3)	O4—C21—H21B	109.9
C8—C9—C10	121.5 (3)	C22—C21—H21B	109.9
C4—C9—C10	121.1 (3)	H21A—C21—H21B	108.3
O4—C10—C11	110.00 (19)	C21—C22—Cl1	112.7 (2)
O4—C10—C9	113.2 (2)	C21—C22—H22A	109.0
C11—C10—C9	111.5 (2)	Cl1—C22—H22A	109.0
O4—C10—C1	105.56 (18)	C21—C22—H22B	109.0
C11—C10—C1	113.6 (2)	Cl1—C22—H22B	109.0
C9—C10—C1	102.83 (19)	H22A—C22—H22B	107.8
			
C3—O2—C2—C1	−101.1 (3)	C2—C1—C10—O4	−59.2 (3)
O1—C1—C2—O2	−168.3 (3)	O1—C1—C10—C11	11.1 (3)
C10—C1—C2—O2	22.2 (4)	C2—C1—C10—C11	−179.8 (2)
O1—C1—C2—C3	125.0 (3)	O1—C1—C10—C9	−109.5 (3)
C10—C1—C2—C3	−44.4 (3)	C2—C1—C10—C9	59.6 (3)
C2—O2—C3—C4	107.8 (3)	O4—C10—C11—C12	112.0 (3)
C1—C2—C3—O2	112.0 (3)	C9—C10—C11—C12	−14.3 (3)
O2—C2—C3—C4	−107.5 (3)	C1—C10—C11—C12	−129.9 (3)
C1—C2—C3—C4	4.5 (4)	O4—C10—C11—C20	−61.6 (3)
O2—C3—C4—C5	132.5 (3)	C9—C10—C11—C20	172.0 (2)
C2—C3—C4—C5	−159.9 (3)	C1—C10—C11—C20	56.4 (3)
O2—C3—C4—C9	−53.1 (4)	C20—C11—C12—O3	175.6 (2)
C2—C3—C4—C9	14.5 (4)	C10—C11—C12—O3	1.8 (4)
C9—C4—C5—C6	0.4 (5)	C20—C11—C12—C13	−4.5 (4)
C3—C4—C5—C6	174.8 (3)	C10—C11—C12—C13	−178.2 (3)
C4—C5—C6—C7	−2.2 (6)	C8—O3—C12—C11	11.4 (4)
C5—C6—C7—C8	1.0 (6)	C8—O3—C12—C13	−168.5 (3)
C12—O3—C8—C9	−9.9 (4)	C11—C12—C13—C14	0.3 (5)
C12—O3—C8—C7	167.8 (3)	O3—C12—C13—C14	−179.7 (3)
C6—C7—C8—O3	−175.5 (3)	C12—C13—C14—C15	1.2 (6)
C6—C7—C8—C9	2.2 (5)	C13—C14—C15—C16	179.6 (3)
O3—C8—C9—C4	173.6 (3)	C13—C14—C15—C20	1.5 (5)
C7—C8—C9—C4	−3.9 (4)	C14—C15—C16—C17	−176.8 (4)
O3—C8—C9—C10	−4.4 (4)	C20—C15—C16—C17	1.4 (6)
C7—C8—C9—C10	178.1 (3)	C15—C16—C17—C18	1.5 (6)
C5—C4—C9—C8	2.7 (4)	C16—C17—C18—C19	−1.8 (6)
C3—C4—C9—C8	−171.7 (3)	C17—C18—C19—C20	−0.7 (5)
C5—C4—C9—C10	−179.4 (3)	C18—C19—C20—C15	3.5 (4)
C3—C4—C9—C10	6.3 (4)	C18—C19—C20—C11	−176.6 (3)
C21—O4—C10—C11	−67.8 (3)	C14—C15—C20—C19	174.5 (3)
C21—O4—C10—C9	57.6 (3)	C16—C15—C20—C19	−3.8 (4)
C21—O4—C10—C1	169.3 (2)	C14—C15—C20—C11	−5.4 (4)
C8—C9—C10—O4	−108.7 (3)	C16—C15—C20—C11	176.3 (3)
C4—C9—C10—O4	73.4 (3)	C12—C11—C20—C19	−173.0 (3)
C8—C9—C10—C11	15.9 (3)	C10—C11—C20—C19	0.7 (4)
C4—C9—C10—C11	−162.0 (2)	C12—C11—C20—C15	6.9 (4)
C8—C9—C10—C1	137.9 (2)	C10—C11—C20—C15	−179.4 (2)
C4—C9—C10—C1	−40.0 (3)	C10—O4—C21—C22	171.6 (2)
O1—C1—C10—O4	131.7 (2)	O4—C21—C22—Cl1	−62.9 (3)

### Hydrogen-bond geometry (Å, °)

**Table:**

*D*—H···*A*	*D*—H	H···*A*	*D*···*A*	*D*—H···*A*
C14—H14A···O2^i^	0.95	2.55	3.392 (4)	147
C19—H19A···O4	0.95	2.54	3.084 (3)	117

Symmetry codes: (i) *x*, *y*+1, *z*.
